# Influence of biofertilizer on heavy metal bioremediation and enzyme activities in the soil to revealing the potential for sustainable soil restoration

**DOI:** 10.1038/s41598-023-44986-8

**Published:** 2023-11-24

**Authors:** Mohammed Haroun, Shifan Xie, Waleed Awadelkareem, Juanjuan Wang, Xiaoqing Qian

**Affiliations:** 1 Department of Agriproduct and Environmental Safety, College of Agriculture, Yangzhou University, Yangzhou, 225012 China; 2 Department of Biotechnology, Africa City of Technology, Khartoum, 11111 Sudan; 3 Ministry of Agriculture and Rural Affairs, Key Laboratory of Cultivated Land Quality Monitoring and Evaluation, Yangzhou University, Yangzhou, 225127 China; 4 Environment Science and Engineering College, Yangzhou University, Yangzhou, 225127 China; 5 Department of Botany, College of Bioscience and Biotechnology, Yangzhou University, Yangzhou, 225009 China; 6 Department of Soil Science, College of Agriculture, Red Sea University, Port Sudan, 33319 Sudan

**Keywords:** Biochemistry, Biotechnology, Biogeochemistry, Environmental sciences

## Abstract

Overuse of chemical fertilizer and pesticides in agricultural activity is frequently damaging to soil health and can accumulate heavy metals in the soil environment, causing harm to plants, humans, and the ecosystem. This study was done to evaluate the effectiveness of biofertilizers in reducing heavy metal levels in contaminated soil and enhancing the activity of soil enzymes that are crucial to plant growth and development. Two bacteria strains, *Pseudomonas aeruginosa*. and *Bacillus firmus*, were chosen to develop biofertilizers based on molasses. The pot experiment was setup using a completely randomized design with four treatments and five levels; *Bacillus firmus* and *Pseudomonas aeruginosa* were used separately, and they were combined for the biofertilizer dose (20, 40, 60, 80, and 100 mL). Utilizing contaminated soils taken from a greenhouse farm the effect of biofertilizer on heavy metal bioremediation and soil enzyme activity was examined. Methods of soil agrochemical analysis were used to determine the soil physiochemical properties and the concentrations of heavy metals Cu, Fe, Zn, Cd, Mo, Mn, were determined by inductively coupled plasma–mass spectrometry ICP-MS, following DTPA extraction methods. In results, soil pH decreased from 8.28 to 7.39, Ec increased from 0.91 to 1.12, organic matter increased from 18.88 to 20.63 g/kg, N increased gradually from 16.7 to 24.4 mg/kg, and K increased from 145.25 to 201.4 mg/kg. The effect of biofertilizer treatment on soil physiochemical characteristics was significantly positive. Application of biofertilizer significantly increased the heavy metal bioavailability and the activities of soil enzymes. Soil pH were positively correlated with soil Zn (0.99819*), APK (0.95869*) activity and negatively correlated with Fe (0.96759*) also statistically significant at (*p* < 0.05). The soil Cu positively correlated with Fe (0.99645*), Cd (0.97866*), β.D.GLU (0.99769*) and negatively correlated with PAK (− 0.9624*). Soil ARY had positive correlation with soil Mn (0.99683*), Cd (0.95695*), and negative correlation with PAK (− 0.99424*) at (*p* < 0.05). Soil enzyme activities were negatively correlated to heavy metals at a significant level. Collectively, the study highlights the potential of biofertilizers as a sustainable and effective approach to enhance soil health and remediate heavy metal-contaminated soils in greenhouses.

## Introduction

Biofertilizers sustain agricultural land’s soil structure and biodiversity and are eco-friendly, cost-effective, non-toxic, and simple to apply. This makes them an effective alternative to chemical fertilizers^1,2^. Biofertilizers, also known as microbial inoculants, are organic preparations containing particular microorganisms from plant roots and root zones. They increase the plant’s growth and yield by 10 to 40%^3^. When applied to the seed, plant surface, or soil, these bioinoculants colonize the rhizosphere and the inside of the plant, which helps the plant grow^4^. They supply nutrients to the soil and protect the plant from pests and diseases, improving soil fertility and crop yield^2^. Another benefit is that biofertilizers are no longer needed after 3 to 4 years since parental inoculants are enough for reproduction^5^. Biofertilizers increase agricultural output and soil fertility and they increase soil structure, crop production, and nutrient cycling when applied to soil^6–8^. The use of potential biofertilizers will improve soil efficiency and sustainability, reduce agricultural pollution, and improve food quality^9^. Biofertilizers dissolve the key nutrients and make them available to the plants^10,11^. Biofertilizers improve plant nutrition and stress tolerance by fixing atmospheric nitrogen and solubilizing soil nutrients^12,13^. Field studies have revealed that the yield of food crops can be increased by approximately 25% applying biofertilizers while reducing the use of nitrogenous and phosphatic fertilizers by about 25–50% and 25%, respectively^14^.

Heavy metals (HMs) are a common cause of soil pollution all over the world. HMs pollutants in the soil have become a major concern due to their toxic effects on human health and the environment^15^. These pollutants are mainly introduced into the soil through human activities such as mining, use of agrochemicals, burning of fossil fuels, industrial waste discharge, and waste disposal^16,17^. High levels of HMs are directly toxic because they stop intracellular enzymes from working and cause oxidative stress, which damages cellular structures^18^. Accumulated zinc (Zn), cadmium (Cd), copper (Cu), manganese (Mn), and iron (Fe) are considered to be major pollutants in soil and water, and these metals are not readily degraded into useful chemicals^19,20^. Farmers worldwide are now focusing on the protection of decreasing agricultural land and the restoration of resources to their pristine condition in response to the increasing issue of soil contamination^21^. Changes in the physical, chemical, and biological properties of the soil and an increase in secondary contamination are also big problems. Due to these issues, physicochemical approaches have been considered unable to be applied in agriculture.

Various HMs resistant microorganisms have recently been identified by researchers from polluted areas, mine dumps and abandoned sites, industrial, waste dumping yards, and the rhizosphere of plants growing in HMs-contaminated soil^22,23^. The isolated bacterial genera, such as *Arthrobacter, Enterobacter, Corynebacterium, Stenotrophomonas, Bacillus*, and *Pseudomonas* play an important role in the bioremediation process. *Bacillus*-based biofertilizers are more active than *Pseudomonas*-based ones because *Bacillus* spp. produces more metabolites and forms spores, which improves cell viability in commercially prepared products^24,25^. *Bacillus*-based biofertilizers increase plant-available nutrients in rhizospheres, reduce pathogenic microbe development, and activate pest defense mechanisms^26^. Further, the microbial communities in these contaminated soils are disrupted, which affects their important roles in recycling organic matter, controlling plant diseases, boosting plant growth, and getting clear of harmful chemicals in the soil^27,28^. The inoculation of *Bacillus* spp. into heavy metal-contaminated soil can reduce the harmful effects of these metals on plant growth through support increasing water uptake and reducing electrolyte to reduce Cd stress^29^.

Due to the viability and efficiency of utilizing bacteria in bioremediation, especially the use of bacteria to remove HMs from contaminated soil, attention has been drawn to this strategy. Bacteria have several ways to process HMs through general resistance mechanisms, bioremediation, and efflux mechanisms. Lead (Pb), chromium (Cr), arsenic (As), zinc (Zn), cadmium (Cd), copper (Cu), mercury (Hg), and nickel (Ni) are the most common HMs. Unlike other pollutants, HMs are mostly stored in the soil. At high concentrations, these contaminants are very harmful to both plants and microorganisms^30^. Bioremediation encompasses all biological approaches for pollution reduction. Bioremediation is a potential approach to clean up polluted soil economically and sustainably^31^. Bioremediation uses plants, animals, and microbes to remove contaminants such heavy metals. It is one of the most effective, non-invasive, and economically viable strategies for substantially reducing heavy metal contamination and restoring many ecosystems’ natural environments^32^. As a consequence, bioremediation has been widely accepted and recommended as a modern technology to resolve the issues correlated with other remediation methods to remove contaminants from the soil in an eco-friendly and sustainable approach^33,34^. The future profitability of bioremediation with biofertilizers such as nitrogen fixing organisms and nutrient mobilizers including phosphate, potassium, and zinc solubilizers, iron sequesters, and sulfur oxidizers, on the other hand, is dependent on biological considerations like competence and ability to effectively remove the stated or native biofertilizers and abiotic factors such as nutrients, pH, and temperature^35,36^.

Microorganisms and plant or animal wastes are the main sources of soil enzymes. Enzymes accumulate in soil as free enzymes or stabilized on clay surfaces or soil organic matter^37^. Furthermost enzymes that are regularly used to assess the impact of heavy metals pollution may be separated into two groups: oxidoreductases [such as dehydrogenase (DH)] and hydrolases [such as β-D-glucosidase (β-D-GLU), phosphatase (PHO), urease (URE) and arylsulfatase (ARYL)]^38^. HMs concentration and soil enzyme activity are usually negatively correlated. Thus, soil enzyme investigations have been used to identify HM contamination in soils^39,40^. Bioavailability of HMs are the most important factor in microbial processes and enzyme activity. Extensive research have established that the biological impacts of contaminants are not connected to the overall concentration of a contaminant in soils^41,42^. Alternatively, organisms also respond to the part of the signal that is biologically available to them. ISO 11074^42^ defines the concept of bioavailability as follows: “Bioavailability is the degree to which chemicals in the soil can be absorbed or decomposed by humans or other organisms, or can interact with biological systems. However, according to Lee et al.^43^, soil enzyme (DH, PHO, and UR) activity are significantly correlated with soil physio-chemical characteristics.

Molasses is the most commonly applied organic carbon substrate in biofertilizer^44^. Indeed, molasses is used to help bacteria generate anaerobic reducing conditions as part of the bioremediation process^45^. Briefly explained, molasses provides more organic carbon to soil bacteria, allowing them to develop and remove contaminants from the soil^46^. Considering organic carbon is the most essential building material for bacteria, it may be the most visible and valuable component of biofertilizer^47^.

The chinese celery cabbage (*Brasica rapa var chinensis* L.) it’s one of the most economically important vegetable families in the world. However, this vegetable has high sensitive to heavy metal concentration in the soil.

In this study, we hypothesized that (I) to investigate the influence of biofertilizers on heavy metal bioremediation and enzyme activities in the soil. (II) to determine if biofertilizers can successfully remediate heavy metal-contaminated soil and restore its fertility in a sustainable manner. Specifically, (III) to assess the potential of biofertilizers in enhancing the degradation and immobilization of heavy metals in the soil, as well as their effects on soil enzyme activities, (IV) to evaluate the effectiveness of biofertilizers on the soil physiochemical properties, and their correlation between soil heavy metal and enzyme activities.

## Materials and methods

### Site description and soil sample collection

This experiment used greenhouse farm soil from the Sha Tou region of Yangzhou city (32°16′23″ N, 119°31′48″ E). Bulk soil samples collected randomly with hand auger from 0 to 20 cm depth was air-dried at room temperature for 14 days. Dry samples were homogenized and sieved (< 2 mm) for future use.

### Pot experiment

The pot experiment was done under greenhouse conditions, during summer season (May 2022 to July 2022) to investigate the impact of biofertilizer on plant growth and heavy metal bioremediation on Chinese celery cabbage growth. The study was designed as factorial arranged in complete randomized design (CRD) with four treatments and three replications. Treatments were four different biofertilizer rates including control treatment, *Bacillus firmus* (20, 40, 60, 80, and 100 mL/ pot), *Pseudomonas aeruginosa* (20, 40, 60, 80, and 100 mL/ pot), and a combination of *Bacillus firmus* and *Pseudomonas aeruginosa* (20, 40, 60, 80, and 100 mL/ pot) respectively. The pot dimensions were measured at 30 cm in height, 20 cm in diameter at the top, and 16 cm in diameter at the bottom. 10 kg of contaminated clay loam soil from the top layer (0–25 cm) was added to the pot. At a depth of 1 cm, five seeds were spread. The soil was sampled in four different treatments following harvest. Each treatment's soil was collected and well mixed before being taken for analysis.

### Determination of soil physiochemical properties

The soil samples were analyzed for pH (1:1 in water) using a pH meter (Shanghai Leici). The soil electrical conductivity (EC) of 1:25 (w/v) in water was measured with an EC meter (TZS-EC-I; Zhejiang Top Instrument Co., Ltd., Hangzhou, China). The soil total nitrogen (TN) and available nitrogen (N) were measured following the semi-micro Kjeldahl method^48^. Sodium bicarbonate extraction and the molybdenum-antimony resistance colorimetry method were used to determine soil available phosphorus (P) content^49^. Soil available potassium (K) and sodium (Na^+^) were determined by potassium dichromate-external heating with a film photometry (WGH-Shanghai) method^50^. The soil organic matter (O.M) was determined using dichromate oxidation methods. The total phosphorus (TP) was determined using a UV-spectrophotometer (MTHSH, UV-5800PC, Shanghai, China) after being extracted by H_2_SO_4_-HClO_4_. The Olsen-P was extracted with a NaHCO_3_ solution containing 0.5 mol L^−1^ and measured at 710 nm with the molybdenum blue method. The exchangeable Ca2^+^, Mg2^+^, K^+^, Na^+^, and NH_4_^+^ were measured at pH 7 with 1 M ammonium acetate. The concentration of soil-available sulfur (S) was determined by the spectrophotometric method^51^. For all soils, physiochemical properties determinations were performed according to soil agrochemical analysis methods^52^.

### Determination of the soil heavy metals concentration

After spending 24 h in a 5% (v/v) nitric acid solution, all glass and plastic containers were rinsed with ultrapure water and stored for use. 20.00 g of air-dried soil passing through 2 mm screen was placed in a 250 mL Erlenmeyer flask, combined with with 40 mL of DTPA extraction at 25 ± 2 °C, and was thoroughly shaken. Oscillate at a frequency of 180 rpm/min ± 20 rpm/min for 2 h and filter to determine. ICP-MS inductively coupled plasma–mass spectrometry was used to conduct a heavy metal analysis on the dried samples according to standard operating protocols^53^. In basic terms, samples were digested in a solution of HNO_3_-HClO_4_ (80/20, v/v) and Cu, Fe, Zn, Cd, Mo, and Mn concentrations were determined following DTPA extraction methods^54^. Each batch sample's analysis included method blanks, duplicate samples, and soil standard samples (GBW07456, Geophysical and Geochemical Exploration Institute of the Chinese Academy of Geological Sciences) for quality assurance and control. Using DTPA solution, the instrument's absorbance was calibrated to the spectrometer to zero point. The properties of the initial soil analysis were described in Table 1.

**Table 1:** Initial analysis of soil physiochemical properties and heavy metals.

pH	Ec	N	P	K	O.M	TN	TP	Ca+	Mg^2+^	NH^4+^	s	Cu	Zn	Mn	Fe	Mo	Cd
	dsm^−1^	mg/kg	mg/kg	mg/kg	g/kg	mg/kg	mg/kg	mg/kg	mg/kg	mg/kg	mg/kg	mg/kg	mg/kg	mg/kg	mg/kg	mg/kg	mg/kg
8.1	0.289	0.17	1.24	1.00	12.71	0.23	0.72	225	126	6.30	9.5	3.22	9.26	22.07	25.43	0.196	0.33

### Determination of soil enzyme activities

Urease activity was measured using urea as a substrate, as described by Kandeler and Gerber^55^. 5 g of moist soil contained 20% of water content were incubated for 24 h at 37 °C with 1 mL of methylbenzene, 10 mL of 10% urea, and 20 mL of citrate buffer (pH 6.7). Then, 1 mL of filtered soil solution, 1 mL of sodium phenolate, and 3 mL of sodium hypochlorite were added and diluted to 50 mL, and absorbance was determined at 578 nm using a spectrophotometer (MTHSH, UV-5800PC, Shanghai, China). For dehydrogenase activity, iodonitrotetrazolium formazan (INTF) was used as a substrate^56,57^, whereby 1 g of moist soil was mixed with 100 mL of INTF (0.2% *w*/*v*) solution and incubated for 24 h at 30 °C. After incubation, 40 mL of acetone was added, and absorbance was determined at 464 nm. The analysis of alkaline phosphatase activity was performed as stated by Tabatabai and Bremner^58^. The P-nitrophenyl phosphate (p-NPP) was used as the substrate. 1 g of moist soil contained 20% of water content were mixed with 20 mL of 100 mM p-NPP in acetate buffer (pH 5.2) and incubated at 30 °C for 30 min. After incubation, 1 mL of CaCl_2_ and 4 mL of 0.5 M NaOH were added to terminate the reaction and the absorbance was measured at 405 nm. For the β-D-glucosidase activity, nitrophenyl-β-D-glucoside (PNG) was used as a substrate. Briefly, 1 g of soil was mixed with 0.2 mL toluene, 4 mL modified universal buffer (pH 6), and 1 mL PNG solution (25 mM) and incubated for 1 h at 37 °C as previously descried by Eivazi and Tabatabai^59^. After incubation, 1 mL of CaCl_2_ solution and 4 mL of Tris buffer (pH 12) were added, and the absorbance was measured at 405 nm. The activity of arylsulfatase was tested using p-nitrophenyl sulfate solution (p-NSS) as described by Tabatabai and Bremner^60^. 1 g of moist soil was mixed with 1 mL of p-NSS sulfate solution (0.05 M) and incubated for 24 h at 30 °C. After incubation, 1 mL of CaCl_2_ 0.5 M and 4 mL of NaOH 0.5 M were added and the absorbance was measured at 420 nm.

### Production and preparation of the biofertilizer

*Bacillus firms* and *pseudomonas aeruginosa* were cultured in different broths: nutrient broth and beef extract peptone medium broth. 250 mL of each medium was placed into a 500 mL Erlenmeyer flask and autoclaved for 20 min^61^. Before use, one agar plate of each pure bacterial culture was suspended in 10 mL sterilized 0.85% sodium chloride. Liquid medium was inoculated with 0.1% pure bacterial culture, shaken at 115 rpm, and incubated at 30 °C for 3 days. The media that increases the spores of *Bacillus firmus* and *Pseudomonas* spp. cells will be used in the pot experiment.

#### Growth of *Bacillus firmus* and *Pseudomonas aeruginosa*

Bacteria were grown individually in molasses, contain between 0.1% and 2.5% of the mother liquid inoculant was mixed with 1000 mL of molasses-based liquid media in a 2 L Erlenmeyer flask at room temperature for 72 h on a 115 rpm gyratory shaker. The *Bacillus firmus* and *pseudomonas aeruginosa* liquid inoculants were then mixed at a volume ratio of 1:10 for the final volume of 100 mL. *Bacillus firmus* spore and *Pseudomonas aeruginosa* cell counts were performed on nutrient agar for *Bacillus firmus* and beef extract peptone medium broth for *Pseudomonas aeruginosa* at 10, 8 by serial dilution plate method^62^. The optimal composition was stored at room temperature for 28 days for the determination of acidity, electrical conductivity, *E. coli* and *Salmonella* population, and phytohormone.

#### Application of biofertilizer

Molasses, *Bacillus firmus*, and *Pseudomonas aeruginosa* were among the organic substances used to produce liquid biofertilizers. To estimate the approximate concentration of heterotrophic bacteria (4 × 10^5^/mL), a serial dilution was carried out using the supernatant. The dilutions were then plated. This estimated concentration was utilized to adjust the concentration of the biofertilizer in order to standardize the final concentration of biofertilizer treatments for implantation (4 × 10^5^/mL). Each treatment got a different amount of biofertilizer at five levels: 20, 40, 60, 80, and 100 mL of prepared biofertilizer on the first day after planting chinese celery cabbage. Molasses was obtained from a sugar factory. The raw materials were analyzed for pH, Ec, total nitrogen (TN), total phosphorus (TP), and total potassium (TK), organic matter, organic carbon (O.C), and the C/N ratio. The chemical properties of the initial substrate, molasses, were descript in Table 2.

**Table 2:** The biochemical characteristics of the original substrates molasses and biofertilizer composition.

Biochemical Properties	Value	Unit
pH	6.7	
Ec	0.8	dsm^−1^
Sugar content	49	%
Total N	0.6	g/kg
Total P	0.2	g/kg
Total K	0.3	g/kg
O.M	89	g/kg
O.C	43	%
C/N	35	%
*E. coli*	No	
*Bacillus firmus*	4 × 10^5^	CFU
*Pseudomonas aeruginosa*	4 × 10^5^	CFU
Combined BP	4 × 10^5^	CFU

### Statistical analysis

The statistical analysis was performed using origin lab pro software 2021^63^. All experiments were performed in triplicate. Error bars on graphs show the standard error. Pearson correlation significance was calculated among various soil HMs concentrations parameters and soil enzyme activity at 95% confidence. The data were analyzed by analysis of variance (ANOVA) and the means were compared using the Fisher LSD test (*p* < 0.05).

## Results

### Effects of biofertilizer on the soil physiochemical properties

Biofertilizer treatments resulted in significant changes in the physiochemical properties of the soil. The effects of biofertilizer treatments on soil pH levels were significant at *p* < 0.05. Biofertilizer treatments reduced pH levels (Table 3). While the greatest soil pH value was observed in the B treatment, the pH was decreased from (8.28) in the Ck treatment to (7.39) in the B treatment. Biofertilizer treatments increased soil Ec levels (Table 3). The greatest Ec value (1.12 ds m^−1^) was obtained from combined BP treatment, and the lowest Ec value (0.91 ds m^−1^) was obtained from Ck treatment without biofertilizer. The effects of biofertilizer treatments on total N levels were found to be non-significant at *p* < 0.05 (Table 3). Increased available N contents were observed with biofertilizer treatments (Table 3). While the highest available N content (24.4 mg/kg) was observed in combined (BP) treatments, the lowest available N content (16.7 mg/kg) was observed in CK treatments. The effects of biofertilizer treatments on available P contents were significant at *p* < 0.05 (Table 3). While the highest P content (51.72 mg/kg) was observed in B treatment, the minimum P content (35.06 mg/kg) was observed in BP treatment (Table 3). Biofertilizer treatments had a significant at *p* < 0.05 effect on available K levels (Table 3). Biofertilizer treatments increased the available K contents of the soils. The greatest K content (453.3 mg/kg) was obtained from BP treatment, and the lowest value (125.5 mg/kg) was obtained from CK treatment. The effects of biofertilizer treatments on soil organic matter content were significant at *p* < 0.05 (Table 3). Increasing O.M levels were observed with biofertilizer treatments (Table 3). The highest O.M level (20.63 g/kg) was obtained from BP treatments, and the lowest O.M level (15.19 g/kg) was observed in B treatments. The effects of biofertilizer treatments on total N levels were found to be non-significant at *p* < 0.05 (Table 3). A significant effect was found on total P between Ck and treatment at *p* < 0.05 (Table 3).

**Table 3:** The effect of biofertilizer application on the soil physiochemical parameters.

Treatments	pH	Ec dsm^−1^	N mg/kg	P mg/kg	K mg/kg	O.M g/kg	TN g/kg	TP g/kg
Ck	8.28 ± 0.11^a^	0.91 ± 0.10^c^	16.7 ± 1.43^c^	44.24 ± 1.70^ab^	125.5 ± 7.60^b^	18.88 ± 1.07^b^	0.23 ± 0.02^a^	0.77 ± 0.01^b^
B	7.39 ± 0.7^c^	0.99 ± 0.06^b^	21.4 ± 2.25^b^	51.72 ± 3.70^a^	438.1 ± 30.44^a^	15.19 ± 0.71^c^	0.22 ± 0.00^a^	0.84 ± 0.02^a^
P	7.41 ± 0.12^c^	0.98 ± 0.07^b^	18.3 ± 1.26^c^	41.94 ± 4.76^b^	424.6 ± 20.11^a^	18.10 ± 0.79^b^	0.22 ± 0.00^a^	0.81 ± 0.01^ab^
Combined BP	7.68 ± 0.03^b^	1.12 ± 0.05^a^	24.4 ± 0.84^a^	35.06 ± 2.16 ^b^	453.9 ± 21.35^a^	20.63 ± 0.59^a^	0.23 ± 0.02^a^	0.85 ± 0.02^a^
LSD ≥ 0.05	0.0013	1.02	1.80	0.01	2.48	2.31	0.83	0.006

Each value are mean ± ESM, n = 3; Means that do not share a letter are significantly different; Initial letters indicate significant different between treatments; Ck, Control, B, *Bacillus firmus*, P, *Pseudomonas aeruginosa*, BP, combined biofertilizer between *Bacillus firms* and *Pseudomonas aeruginosa.*

### Effects of biofertilizer application on the soil exchangeable nutrients

The impact of biofertilizer application on the soil exchangeable nutrient Ca^2+^, Mg^2+^, K^+^, Na^+^, NH_4_^+^ and sulfur (S) was significantly different were compared between treatments and control, and interaction between dose and treatments except for Ca^2+^ was non-significantly different between treatments and dose. The soil Na^+^ and K^+^ contents were increased by the application of biofertilizers. The effects of biofertilizer and treatments on exchangeable Ca^2+^ levels were non-significant at *p* < 0.05 (Table 4). Treatments with biofertilizer had a significant effect on exchangeable Mg^+^ contents at *p* < 0.05 (Table 4). The highest Mg^2+^ content (73.66 g/kg) was obtained from combined BP treatment, and the lowest Mg^+^ content (61.77 g/kg) was obtained from Ck treatment (Table 4). Biofertilizer treatments had a significant at *p* < 0.05 effect on exchangeable K^+^ contents (Table 4). Exchangeable K^+^ contents increased with biofertilizer treatments (Table 4). The highest K^+^ content (201.50 g/kg) was obtained from combined BP treatment, and the lowest K^+^ content (145.25 g/kg) was obtained from Ck treatment. The effects of biofertilizer treatments on exchangeable Na^+^ contents were found to be significant at *p* < 0.05 (Table 4). The greatest Na^+^ content (230.25 g/kg) was obtained from combined BP treatment, and the lowest Na^+^ content (65.75 g/kg) was obtained from Ck treatment (Table 4). Table 4 shows that biofertilizer treatments had a significant effect on ammonium nitrogen NH_4_^+^ at *p* < 0.05 and soil S at *p* < 0.05.

**Table 4:** The effects of biofertilizer application on the soil exchangeable nutrients.

Treatments	Ca^2+^ g/kg	Mg^2+^ g/kg	K^+^ g/kg	Na^+^ g/kg	NH_4_^+^ g/kg	S g/kg
Ck	425.82 ± 56.89^a^	61.77 ± 5.82^b^	145.25 ± 3.19^d^	65.75 ± 3.20^d^	4.64 ± 0.32^b^	9.47 ± 1.10^b^
B	419.77 ± 27.08^a^	63.60 ± 4.19^b^	169.75 ± 7.78^c^	220.00 ± 18.42^b^	6.38 ± 1.26^a^	10.44 ± 0.60^ab^
P	406.45 ± 39.20^a^	61.79 ± 6.33^b^	179.75 ± 5.75^b^	193.75 ± 15.12^c^	4.93 ± 0.21^b^	12.06 ± 0.94^a^
Combined BP	450.53 ± 24.94^a^	73.66 ± 3.51^a^	201.50 ± 3.07^a^	230.25 ± 12.88^a^	7.21 ± 0.28^a^	11.13 ± 1.05^ab^
LSD ≥ 0.05	0.78	0.04	7.48	6.57	5.94	0.006

Each value are mean ± ESM, n = 3; Means that do not share a letter are significantly different; Initial letters indicate significant different between treatments; Ck. Control, B, *Bacillus firmus*, P, *Pseudomonas aeruginosa*, BP, combined biofertilizer between *Bacillus firmus* and *Pseudomonas aeruginosa.*

### Effects of biofertilizer on the soil heavy metal contents

**Figure 1:**
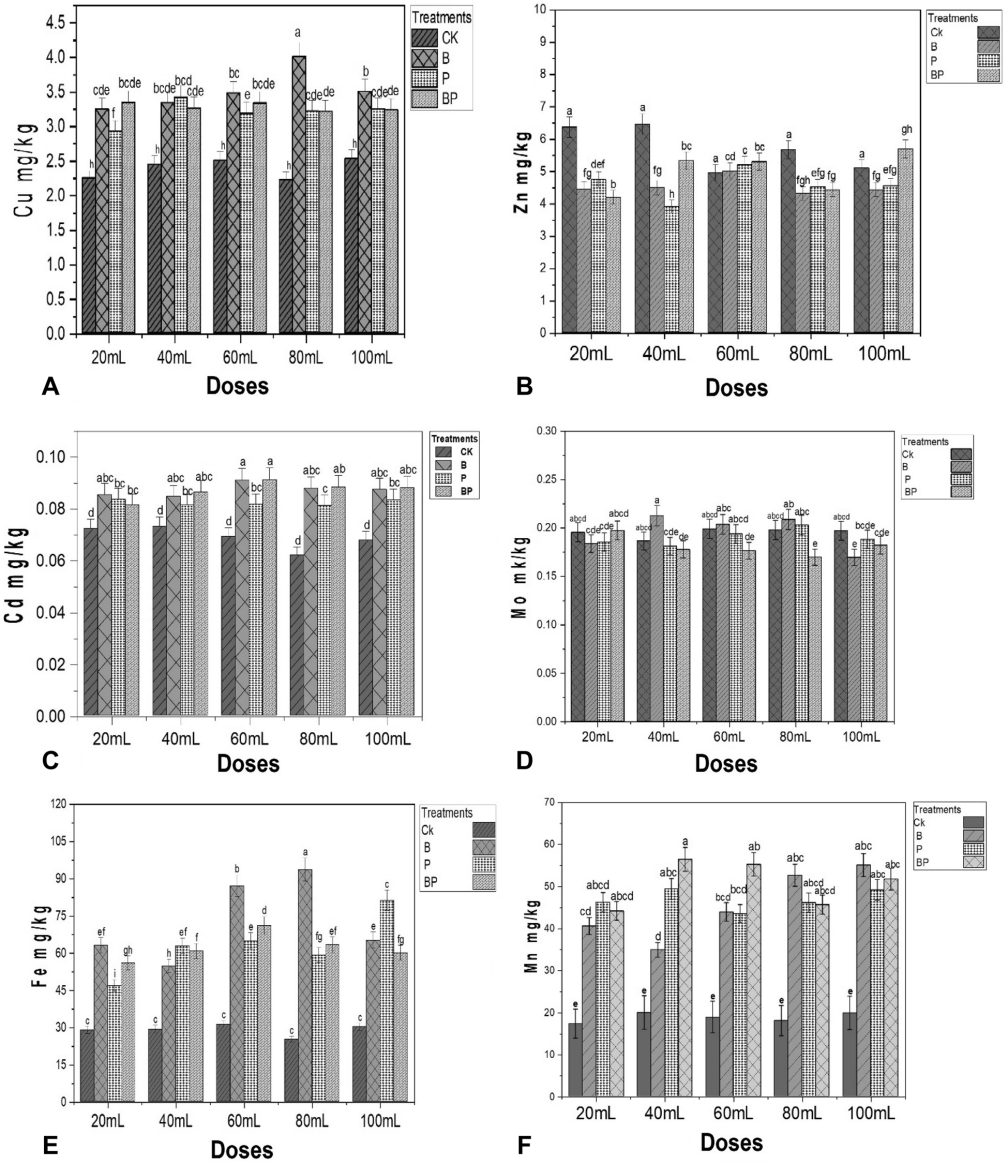
Graphs (**A**) Cu, (**B**) Zn, (**C**) Cd, (**D**) Mo, (**E**) Fe, (**F**) Mn. The interaction between dose and treatments of biofertilizer application, effect of dose concentration (20, 40, 60, 80 and 100/mL) on heavy metal concentration in the soil. After application: (CK) control, (B) *Bacillus firmus*, (P) *Pseudomonas aeruginosa* (BP) combination of *Bacillus firmus* and *Pseudomonas aeruginosa* Values are means ± SE (n = 3). Bars with different letters represent significantly (*p* < 0.05) differences after ANOVA and an LSD (Least significant difference) test.

The application of biofertilizer containing *Pseudomonas aeruginosa* and *Bacillus firmus* was significantly affected by the treatments with different doses of biofertilizer. When compared to the control, the use of biofertilizer treatments and dose levels had a significant effect on Cu bioremediation at *p* < 0.05 (Fig. 1A). Application of biofertilizers on the Zn content were significantly affected by biofertilizer and dose treatments at *p* < 0.05 (Fig. 1B). While the Cd bioremediation content was observed in all treatments with a dose-significant effect compared to the control at *p* < 0.05 (Fig. 1C). Biofertilizer treatments and dose levels did not have clear significant effects on the Mo content at *p* < 0.05 but there is siginificant differences among treatments at *p* < 0.05 (Fig. 1D). Biofertilizer treatments increased soil Zn contents were compared the dose and treatment to control without biofertilizer. The greatest Fe bioremediation level was observed at doses of 80 mL with B treatments and dose 60 mL with B treatments compared to the control (Fig. 1E). While the greatest Mn content was obtained from BP treatment at 40 mL dose compared to the control (Fig. 1F).

### Effects of biofertilizer on the soil enzyme activities

**Figure 2:**
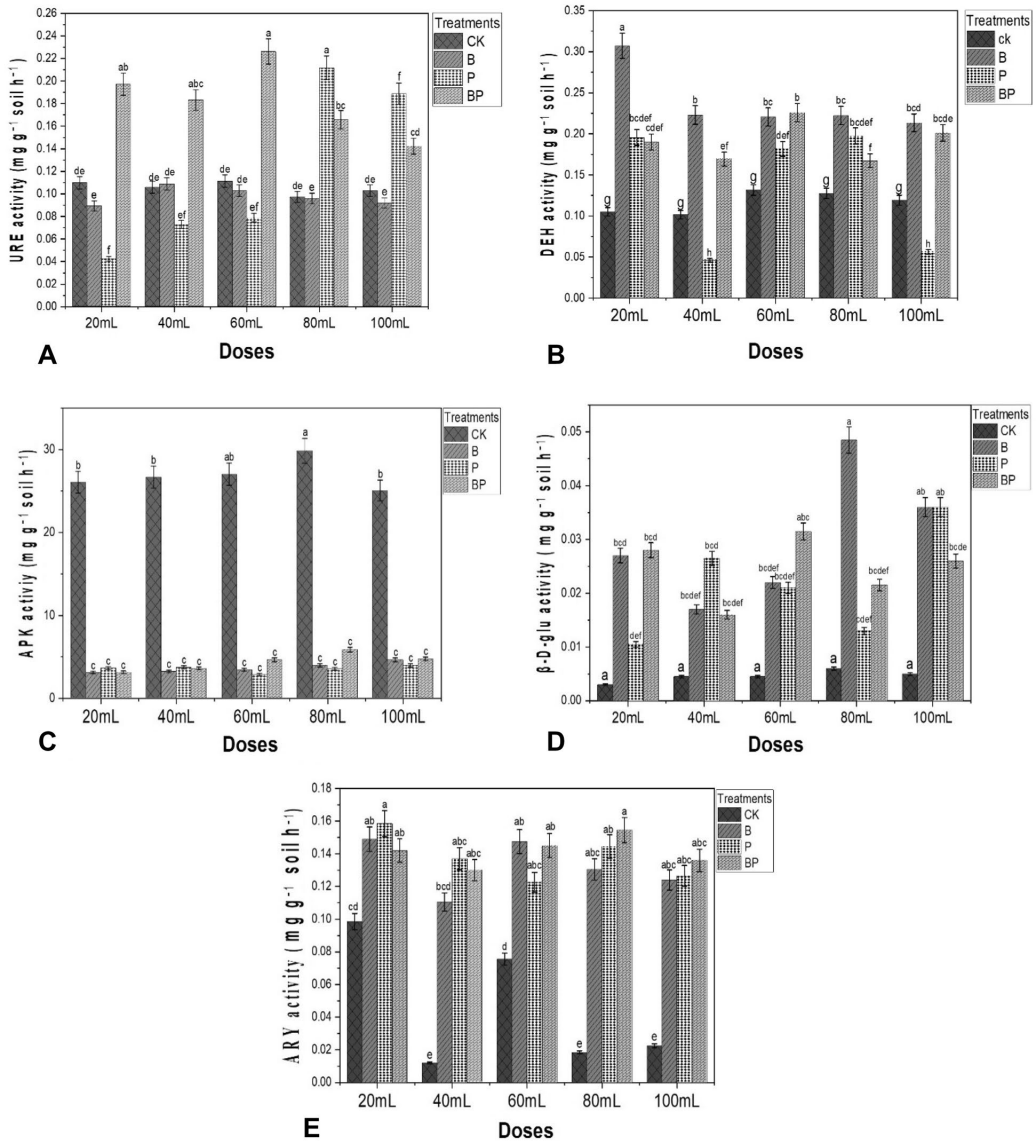
Effects of biofertilizer application on the soil enzyme activity five dose levels (20, 40, 60, 80 and 100/mL) on heavy metal concentration in the soil after application and four treatments represent as (CK) control, (B) *Bacillus firmus*, (P) *Pseudomonas aeruginosa* (BP) combination of *Bacillus firmus* and *Pseudomonas aeruginosa* Values are means ± SE (n = 3). Graphs (**A**) URE, (**B**) DEH, (**C**) APK, (**D**) β-D-GUL, (**E**) ARY. Bars with different letters represent significantly (*p* < 0.05) differences after ANOVA and an LSD (Least significant difference) test.

Soil enzyme activities were determined in the soils treated with CK, B, P, and BP, the influences of different doses of biofertilizer on rhizosphere soil enzyme activities are described in Fig. 2. The soil urease activity was significantly affected at (*p* < 0.05) were compared between treatment and control (Fig. 2A). The application of biofertilizer treatments and dose levels significantly affected on the dehydrogenase activity at (*p* < 0.05) (Fig. 2B). Biofertilizer application was significantly affected on the soil alkaline phosphatase activity at (*p* < 0.05) were compare between treatment and control; but there was no significant difference between treatments or dose levels at (*p* < 0.05) (Fig. 2C). The effect of biofertilizer application on the soil β-D-glucosidase activity was found to be significant at (*p* < 0.05), when compared the treatments and dose levels (Fig. 2D). Furthermore, the effect of biofertilizer application on soil arylsulfatase activity was significant effect at (*p* < 0.05) were compared to control resulted in (Fig. 2E).

### Correlation between soil heavy metal content and enzyme activities

The Pearson correlation coefficients between soil heavy metals and enzyme activity are provided in Table 5. Soil pH were positively correlated with soil Zn (0.99819*), APK (0.95869*) activity and negatively correlated with Fe (0.96759*) also statistically significant at (*p* < 0.05). The soil Cu positively correlated with Fe (0.99645*), Cd (0.97866*), β.D.GLU (0.99769*) and negatively correlated with PAK (− 0.9624*). The URE activity were negatively correlated with Mo (− 0.98885*), pH, PAK and positively correlated with other metals and enzyme activity nevertheless had no significant at (*p* < 0.05). Therefore, soil pH was an important factor among all soil heavy metals and enzyme activity. The negative correlation between soil pH and soil enzyme activity except PAK shows positive correlation with soil pH. However, soil ARY had positive correlation with soil Mn (0.99683*), Cd (0.95695*), and negative correlation with PAK (− 0.99424*) activity. DEH had slight change negatively and positively but did not have any statistically significant relationship with all parameters at (*p* < 0.05). This result indicated that the relationship between soil heavy metals and soil enzyme activities was more closely.

**Table 5:** Pearson correlation analysis between soil heavy metal and soil enzyme activity.

	pH	Zn	Mn	Fe	Cu	Cd	Mo	URE	AKP	β.D.GLU	ARY	DEH
pH	1											
Zn	0.99819*	1										
Mn	− 0.89208	− 0.86331	1									
Fe	− 0.96759*	− 0.95767*	0.92847	1								
Cu	− 0.9429	− 0.93023	0.92865	0.99645*	1							
Cd	− 0.87957	− 0.85521	0.96103*	0.96381*	0.97866 *	1						
Mo	0.16093	0.10192	− 0.58152	− 0.25715	− 0.2889	− 0.46912	1					
URE	− 0.05046	0.00964	0.49451	0.17408	0.21603	0.40836	− 0.98885 *	1				
AKP	0.95869*	0.93991	− 0.98364 *	− 0.97129 *	− 0.9624*	− 0.9575 *	0.42735	− 0.32992	1			
β.D.GLU	− 0.91989	− 0.90669	0.91368	0.98896*	0.99769*	0.98028*	− 0.28539	0.22147	− 0.94462	1		
ARY	− 0.92431	− 0.89968	0.99683*	0.94575	0.94053	0.95695*	− 0.52156	0.42745	− 0.99424*	0.92282	1	
DEH	− 0.62053	− 0.61328	0.61424	0.77983	0.82047	0.80838	− 0.08593	0.09591	− 0.64279	0.85633	0.61097	1

2-talied test of significance is used, *Correlation is significant at the *p* < 0.05 level, Zn, zinc, Mn, Manganese, Fe, Iron, Cu, Coper, Cd, Cadmium, Mo, Molybdenum, URE, Urease, AKP, Alkaline phosphatase, β-D-GLU, β-D-Glucosidase, RAY, Arylsulfatase, DEH, Dehydrogenase.

## Discussion

In this study, particularly *Bacillus firmus* and *Pseudomonas aeruginosa*, which are used as biofertilizers to bioremediate soil, have been polluted with heavy metals due to human activities. This research investigated the effects of biofertilizer on heavy metal bioremediation. These include soil enzyme activity, soil physiochemical parameters, and the correlation between heavy metals in the soil and enzyme activity. Further biofertilizer was applied in five doses (20 mL, 40 mL, 60 mL, 80 mL, and 100 mL) to greenhouse soil grown with Chinese celery cabbage. The results of the current study showed that the application of biofertilizer was directly related to the improvement of soil physiochemical parameters, including soil organic matter and total P. Biofertilizers improve plant nutrition and stress tolerance by fixing atmospheric nitrogen and resolving soil nutrients^12,13^. Biofertilizer promotes soil organic carbon content by releasing organic molecules via the roots^19,64^ found that the organic carbon content of pot soils significantly increased. The current results are consistent with the findings of Faye et al.^65^, who found that using biofertilizer significantly increased the amount of soil organic carbon. Biofertilizer application at five different doses in greenhouse conditions significantly increased the soil available P and K levels in the single treatments, especially P. The soil nutrients were enhanced by low-dose 40 mL and medium-dose 60 mL levels Table 3. These findings demonstrated a direct correlation between the application of *Bacillus firmus* and *Pseudomonas aeruginosa* and their ability to enhance soil nutrient availability. The use of biofertilizer has the potential to increase the amount of P that is easily available in soil since it supports a higher population of a variety of bacteria that are capable of solubilizing soil P^66^. The amount of P in the soil solution may change as root exudates, such as organic ligands, are released^67^. According to literature cited microorganisms primarily solubilize insoluble P by the formation of organic acids and chelating compounds. The use of *Bacillus firmus* and *Pseudomonas aeruginosa* may have resulted in more solubilization of insoluble phosphates in the soil and as a result, increased phosphate uptake^68^. According to Tak et al.^69^ biofertilizer significantly changed ion selectivity, increasing K^+^ and Ca^2+^ uptake while decreasing Na^+^ uptake. It was observed that biofertilizer caused distinct buildup of N, P, and K, therefore maintaining nutritional balance^70,71^. *Bacillus firmus* and *Pseudomonas aeruginosa* have been shown to enhance soil nutrients and consequently fertility in soils^72^. In this study, the soil Na^+^ and K^+^ contents Table 4 were increased by application of biofertilizers.

According to this research, Table 3 soil pH values under greenhouse conditions for Chinese celery cabbage reduced with biofertilizer application, under the five dosage as compared to the treatments without biofertilizer. According to Berger et al.^73^, biofertilizer decreased soil pH and increased soil available P and K levels, due to enhanced K release from organic components and minerals. Numerous parameters, including soil pH and fertility, are associated with the presence of *Bacillus firmus* and *Pseudomonas aeruginosa* in soil^74^. Reference^75^ discovered that biofertilizer inoculation caused a pH shift, slightly lowering alkalinity, and slightly increasing organic matter content in all biofertilizer inoculated treatments compared to uninoculated soil. In this investigation, biofertilizer treatments caused a decrease in soil pH; the overall mean decrease in pH when compared to soils not treated with biofertilizer was 7.71%. The BF + 40 treatment had the lowest pH (7.16). Due to the application of biofertilizer, there was a slight change in the pH of the soil. The pH of the soils decreases as a result of microbial inoculants’ increased amounts of organic acid. The current results are consistent with those of ^76^^,^^77^, who found that applying bio-fertilizers reduced the pH of the soil. These bacteria can produce organic acids as byproducts of their metabolic processes, which can lower the pH of the soil. This acidification can influence the pH levels and make them more acidic.

This research found that biofertilizer treatments significantly affected soil Ec values Table 3, which ranged from 1.79 to 3.29 ds m1. Ec is a major indicator of the soil’s quality and is influenced by the ion concentrations in the soil solution ^78^. As ion concentrations rise, soil Ec values rise as well. The Ec values for soils treated with biofertilizer were higher than among control soils without bio-fertilizers, according to Singh et al.^79^. Reference^80^ reported decreased pH and increased Ec values with bio-compost treatments along with *Azotobacter*. The current research showed that biofertilizer treatments enhanced the amount of cations, particularly Ca^2+^, Mg^2+^, and K^+^. *Azotobacter*-treated plots maintained soil available nutrients and organic carbon concentrations. Our study results showed that the application of biofertilizer increased amount of Mg^2+^, and K^+^ and decrease in the Ca^2+^ at single treatments Table 4, this may be lead to ability of this bacteria to transform the Ca^2+^.

Biofertilizer application improved heavy metals bioremediation in the Cu, Cd, Mo, Fe, and Cd; nonetheless decreased in the Zn at five different doses as compared to treatments without biofertilizer Ck (Fig. 1). Biofertilizers increase heavy metal availability by solubilization, chelation, and oxidation/reduction processes^81^. Biofertilizer develops iron-chelating compounds, reduces soil pH, releases organic acids into the soils, and potentially increases the availability of Fe^75^. Consequently, using biofertilizer and plants for remediation is a potential solution to heavy metal contamination. The application was reduce the Cd and Mo content in the soil^82^, report that the biofertilizer was decrease heavy metals components of Pb, Cd, Mo, in the soil which was extremely intensive for using chemical fertilizer and pesticides. In contrast, the use of biofertilizer increased the bioavailability of Cu in both bacteria strains utilized, *Bacillus firmus* and *Pseudomonas aeruginosa.* This study demonstrated the possible application of biofertilizer in the bioremediation of heavy metals in soil, and a similar study was noted by Mesa-Marín et al.^83^ *S. ramosissima* plant growth was increased by inoculation with heavy metal resistant PGPB. In our investigation, the effect of different doses of biofertilizer was to decrease the levels of Zn in the soil and increase the levels of Mn, which leads to the use of bacteria spices in this study, *Bacillus firmus* and *Pseudomonas aeruginosa*, which may work depending on heavy metal concentration and bacteria mechanisms. According to Abdel-Azis et al.^84^, biofertilizers have the potential to reduce the content of heavy metals in both polluted soil and plant parts. Beyond their impact on enhancing plant development via plant growth promotion, chemicals and bacteria nitrogen fixing (BNF) mechanisms, a similar function of biofertilizer was found. The bacteria existing in biofertilizers have the ability to immobilize heavy metals. This means that they can bind heavy metals to their cellular components, reducing their availability for uptake by plants. As a result, Fig. 1, heavy metal concentrations in the soil was decrease when biofertilizers are used.

According to this study, the application of biofertilizer had a significant effect on the soil enzyme activity and bioremediation of the HMs, Fig. 2 on both the structure and activity. According to Yang et al.^85^, the enzyme activity as stated by Oleszczuk et al.^86^, the toxicological effects of Zn, Cu, Mn, Fe, Mo, and Cd on the structure and activity of soil microbial communities are shown to be highly dependent on concentration of heavy metals content and application duration. Furthermore, soil enzyme activity is a measure of ecosystem health and sustainability^87^. The C, N, and P looping in soil are closely related to, urease, and phosphatase activity^41^. According to the current investigation, applying biofertilizer significantly increased the activity of the soil enzymes urease, dehydrogenase, β-D-glucosidase, arylsulfatase, and when compared to Ck (Fig. 2A,B,D,E) and decrease in the phosphatase were compare to Ck (Fig. 2C). this suggests that applying biofertilizer might raise soil enzyme activities. Similarly report that changes in rhizosphere bacteria activity might explain the increase in enzymatic activities^88^. Many studies mentions that the diversity of native microorganisms may improve during biofertilizer application^89,90^. According to this study, the use of biofertilizer may have contributed to the increase in soil enzyme activity. The use of biofertilizers is considered to promote cabbage growth by increasing the amount of available phosphorus in the soil, modifying the soil community, and activating soil enzymes. Inclusive, these chosen soil enzymes provide valuable evidence about the effects of heavy metal contamination on important soil biochemical processes involved in nutrient cycling, organic matter decomposition, and soil health. They help in assessing the potential for bioremediation strategies to restore the functionality and fertility of soils heavy metal-contaminated.

Heavy metals and soil enzyme activity were both impacted by the correlation between the various soil characteristics^91^. Soil enzymes have a significant role in the fundamental biochemical characteristics of soil. Additionally, soil enzymes influence the formation, composition, and biochemical characteristics of enzymes^92^. The activities of enzymes (acid and alkaline phosphatases, β-D-glucosidase, and arylsulfatase) have been reported to be positively correlated with soil heavy metal concentrations^67^. The outcomes of this investigation were complex. Table 5 shows that Zn was negatively correlated with AKP and URE activity, but the Mn in the soil was positively correlated with URE, AKP, β-D-GUL, ARY, and DEH activity. The activity of the AKP is negatively correlated with the Cu, Fe, and Cd concentrations in the soil. These results indicated that Mo correlated negatively with all soil enzyme activities except for AKP, which positively correlated. Overall, the duration of the study might not capture long-term effects or variations in the bioremediation process and enzyme activities. Different timeframes might lead to different results, as the efficiency of biofertilizer treatment and enzymatic activities could change over time.

## Conclusions

This study was investigated *Bacillus firmus* and *Pseudomonas aeruginosa* as biofertilizers, and their ability to perform heavy metal bioremediation in the soil was confirmed. Positive effects on soil physicochemical porprates, availability of soil macronutrients, exchangeable nutrients, and six heavy metals were observed under greenhouse conditions. When microbial biofertilizer treatments were applied at five different dosage levels (20 mL to 100 mL). Biofertilizer treatments at all dosage levels enhanced soil organic carbon content and aggregate stability considerably as compared to non-fertilizer applications. Biofertilizer treatments had also significantly decreased contributions to the soil pH and Ec. Based on the outcomes of this study, it was determined that biofertilizer might be utilized as an alternative element of integrated nutrient management systems. As a result, it is possible to conclude that soil enzyme activity was increased in the URE, ARY, DEH, β-D-GUL and decreased in the APK during *Pseudomonas aeruginosa* biofertilizer application. The best dose found in the enzyme activities was 80 mL, and heavy metal bioremediation was increased at a dose of 60 mL with *Bacillus firmus* biofertilizer application, which was better than *Pseudomonas aeruginosa* and combination biofertilizer. Furthermore, the use of biofertilizer for bioremediation processes was recommended as a sustainable, particular, and low-cost substitute for heavy metal bioremediation in contaminated soils. In the prospect of using biofertilizer prepared through molasses for contaminated greenhouse soil, we recommended that, based on the findings of this research, 60 mL be used as dose levels with *Bacillus firmus* for heavy metal bioremediation and 80 mL as dose levels with *Pseudomonas aeruginosa*, a combination biofertilizer for soil enzyme activities. There are several potential directions for future research: 1. Optimization of biofertilizer formulations, 2. Assessment of long-term effects, 3. Examination of enzyme mechanisms, 4. Field-scale investigations, 5. Environmental and economic assessments. By addressing these research directions, scientists can further enhance our understanding of the influence of biofertilizer on heavy metal bioremediation and enzyme activities in the soil to revealing the potential for sustainable soil restoration, paving the way for more effective and sustainable soil remediation strategies.

## Data Availability

The datasets generated and analyzed during the current study are available from the corresponding author on reasonable request.

## Abbreviations

AKP: Alkaline phosphatase
β.D. GLU: β-D glucosidase
RAY: Arylsulfatase
DEH: Dehydrogenase
URE: Urease
HMs: Heavy metals
Zn: Zinc
Cd: Cadmium
Cu: Copper
Mn: Manganese
Fe: Iron
Pb: Lead
Cr: Chromium
As: Arsenic
Hg: Mercury
Ni: Nickel
ICP-MS: Inductively coupled plasma–mass spectrometry
